# Elraglusib (9-ING-41), a selective small-molecule inhibitor of glycogen synthase kinase-3 beta, reduces expression of immune checkpoint molecules PD-1, TIGIT and LAG-3 and enhances CD8^+^ T cell cytolytic killing of melanoma cells

**DOI:** 10.1186/s13045-022-01352-x

**Published:** 2022-09-14

**Authors:** Gary Shaw, Ludimila Cavalcante, Francis J. Giles, Alison Taylor

**Affiliations:** 1 School of Medicine, Leeds Institute of Medical Research, University of Leeds, Wellcome Trust Brenner Building, St James’s University Hospital, Leeds, LS9 7TF UK; 2 Winship Cancer Institute of Emory, University School of Medicine, Atlanta, GA USA; 3 Developmental Therapeutics LLC, Chicago, IL USA

**Keywords:** Glycogen synthase kinase-3, Elraglusib, 9-ING-41, Melanoma, T cells, Tbet/Tbx21, PD-1: programmed cell death protein 1, TIGIT: T cell immunoreceptor with immunoglobulin and ITIM domains, LAG-3: lymphocyte activation gene-3

## Abstract

**Background:**

Glycogen synthase kinase-3 (GSK-3) is a serine/threonine kinase with multiple roles in tumour growth, cell invasion and metastasis. We have previously established GSK-3 as an upstream regulator of PD-1 gene expression in CD8 + T cells and demonstrated that GSK-3 inhibition is as effective as anti-PD-1 mAb blockade in controlling tumour growth. Elraglusib (9-ING-41) is a specific small-molecule inhibitor of GSK-3β with clinical activity in patients with advanced cancers, including a patient with refractory melanoma whose response provided the rationale for the current study.

**Methods:**

The B16 melanoma mouse model was used to observe the effect of elraglusib on tumour growth either as a single agent or in combination (simultaneously and sequentially) with anti-PD-1 mAb treatment. B16 tumour cells were implanted in either the flank, brain or both locations, and Kaplan–Meier plots were used to depict survival and significance determined using log rank tests. Expression of the immune checkpoint molecules, TIGIT, LAG-3 and PD-1, was evaluated using flow cytometry alongside expression of the chemokine receptor, CXCR3. Further evaluation of PD-1 expression was determined through RT-qPCR and immunohistochemistry.

**Results:**

We demonstrated that elraglusib has a suppressive effect against melanoma as a single agent and enhanced anti-PD-1 therapy. There was a synergistic effect when elraglusib was used in combination with anti-PD-1 mAb, and an even greater effect when used as sequential therapy. Suppression of tumour growth was associated with a reduced expression of immune checkpoint molecules, PD-1, TIGIT and LAG-3 with upregulation of CXCR3 expression.

**Conclusions:**

These data highlight the potential of elraglusib as an immune-modulatory agent and demonstrate the benefit of a sequential approach with immune checkpoint inhibition followed by GSK-3β inhibition in melanoma and provide a rationale for clinical investigation of elraglusib combined with immune checkpoint inhibitory molecules, including those targeting PD-1, TIGIT and LAG-3. This has several potential implications for current immunotherapy regimes, including possibly reducing the intensity of anti-PD-1 mAb treatment needed for response in patients receiving elraglusib, especially given the benign adverse event profile of elraglusib observed to date. Based on these data, a clinical study of elraglusib, an anti-PD-1 mAb and chemotherapy is ongoing (NCT NCT05239182).

**Supplementary Information:**

The online version contains supplementary material available at 10.1186/s13045-022-01352-x.

## Background

Glycogen synthase kinase-3 (GSK-3), a regulatory kinase first discovered in 1980 to phosphorylate and inhibit glycogen synthase [1], is involved in many cellular processes ranging from glycogen metabolism to gene transcription, apoptosis and microtubule stability [2, 3]. There are two isoforms of GSK-3 reported in mammals: a 51 kDa GSK-3α and a 47 kDa GSK-3β isoform, encoded by the unlinked GSK-3a and GSK-3b genes, respectively. Both isoforms are highly expressed in many tissues [4] and exhibit 98% homology in their kinase domains, but only 36% identity in the last 76 C-terminal amino acid residues. GSK-3 is unusual, in that it is constitutively active in resting cells [1, 4] and is inactivated through phosphorylation of specific serine residues (Ser9 in GSK-3β, Ser21 in GSK-3α) [5, 6]. In its active state, GSK-3 phosphorylates more than 100 substrates [7] and inhibits T cell activation and cytokine production [8–10]. Ligation of the T cell receptor (TCR) and co-stimulatory receptor, CD28, induces phosphorylation of GSK-3 in a phosphatidylinositol 3 kinase (PI3-K)-dependent manner [11] and results in its inactivation [8, 12, 13].

GSK-3β has multiple roles in tumour progression including the modulation of oncogenes, cell cycle regulators and mediators of epithelial–mesenchymal transition [14–16]. Aberrant overexpression of GSK-3β promotes tumour growth and chemotherapy resistance in a spectrum of solid tumours through differential effects on pro-survival NF-κB and c-Myc pathways as well as on TNF-related apoptosis-inducing ligand (TRAIL) and p53-mediated apoptotic mechanisms [4, 6]. GSK-3β is thus a potentially important therapeutic target in human malignancies. We have previously shown that inactivation of GSK-3 with small interfering RNAs (siRNAs) and drug inhibitors enhanced Tbet (*Tbx21*) transcription. This in turn down-regulated expression of the checkpoint molecule programmed death-1 (PD-1) by repressing the *Pdcd1* promoter [9, 11, 17] resulting in enhanced CD8 + cytolytic T cell (CTL) function and the suppression of tumour growth and viral infections [10, 18].

Elraglusib is a reversible ATP-competitive small-molecule inhibitor with significant pre-clinical anti-tumour activity in a broad spectrum of cancers including pancreatic, glioblastoma, neuroblastoma, breast, ovarian, bladder cancer and sarcomas [19–26]. These studies have utilized elraglusib as both a single agent as well as in combination with standard cytotoxic agents [23, 25] or BCL-2 and CDK-9 antagonists [27]. Elraglusib’s clinical activity has also been reported in patients with advanced refractory cancers [28, 29]. In the present study, we evaluate elraglusib in *vitro* and in a B16 melanoma mouse model and compare its efficacy with anti-PD-1 mAb therapy as both a single agent and when combined with PD-1 mAb therapy.

## Methods

### Mice and cells

C57BL/6 J mice were used alongside OT-I Tg mice. Spleen cells were treated with a hypotonic buffer with 0.15 M NH4CL, 10 mM KHCO3 and 0.1 mM EDTA, pH 7.2 to eliminate red blood cells before suspension in RPMI 1640 medium supplemented with 10% FCS, 50uM beta-mercaptoethanol, sodium pyruvate, 2 mM L-glutamine, 100 U/ml penicillin and streptomycin (GIBCO). T cells were isolated from tumour infiltrating cells, spleen and lymph node samples using negative section of magnetically labelled cells (Miltenyi). B16F10-fluc melanoma and EL4 lymphoma cells were cultured in DMEM medium that was supplemented as above. The research was regulated under the Animals (Scientific Procedures) Act 1986 Amendment Regulations 2012 Home Office UK PPL No. P0CFA732A.

### Generation of cytolytic T cells

OVA-specific CD8 + cytolytic T cells (CTLs) were generated by incubating isolated splenocytes OT-I mice with SIINFEKL peptide of OVA257-264 peptide used at 10 nM (Bachem Ag) for 5 days. CTLs were generated in the presence or absence of elraglusib and/or anti-PD-1 for 5 days prior to washing and analysis by FACs, PCR or in cytotoxicity assays. Elraglusib was reconstituted in DMSO to give a stock solution of 25 mM and diluted to a concentration of 10 uM for in vitro assays.

### Cytotoxicity assays

Cytotoxicity was assayed using a Cytotox 96 non-radioactive kit (Promega) following the instructions provided. In brief, purified T cells were plated in 96-well plates at the effector/target ratios shown using 10^4^ EL4 (pulsed with OVA peptide). Target cells per well were in a final volume of 200 µl per well using RPMI lacking phenol red. Lactate dehydrogenase release was assayed after 4 h incubation at 37 °C by removal of 50 µl supernatant from each well and incubation with substrate provided for 30 min and the absorbance read at 490 nm using the Thermomax plate reader (Molecular Devices). Percentage cytotoxicity = ((experimental-effector spontaneous–target spontaneous)/(target maximum–target spontaneous)) × 100. All cytotoxicity assays were reproducible in at least three independent assays.

### Antibodies/reagents

The following antibodies were used in experiments: InVivo Anti-PD-1 (CD279, J43) (BioXCell), Elraglusib (9-ING-41) kindly provided by Actuate Therapeutics Ltd and OVA_257-264_ peptide (Bachem Ag). Conjugated antibodies for flow cytometry: anti-CD8α (clone, 53–6.7), anti-CD3 (eBioscience) and CD279 (clone EH12.2H7) (BioLegend). Antibodies for IHC: anti-PD-1 (Cell Signaling Technology), and anti-CD8 (Abcam).

### Flow cytometry

Flow cytometry of antibody staining of surface receptors was conducted by suspending 10^6^ cells in 100 μl PBS and adding antibody (1:100) for 2 h at 4 °C. Cell staining was analysed on a Beckman Coulter CytoFLEX S flow cytometer and by CytExpert software.

### Quantitative real-time polymerase chain reaction (PCR)

Single-strand cDNA was synthesized with an RT-PCR kit (Qiagen) according to the manufacturer’s instructions. Reverse transcription was performed using the RNA polymerase chain reaction (PCR) core kit (Applied Biosystems). Quantitative real-time PCR used SYBR green technology (Roche) on cDNA generated from the reverse transcription of purified RNA. After preamplification (95 °C for 2 min), the PCRs were amplified for 40 cycles (95 °C for 15 s and 60 °C for 60 s) in a sequence detection system (PE Prism 7000; Perkin-Elmer Applied Biosystems). The exponential phase, linear phase and plateau phase of PCR amplification were carefully monitored to ensure a measurement of real-time transcription [30]. mRNA expression was normalized against GAPDH expression using the standard curve method.

PD-1-FW, 5-CCGCCTTCTGTAATGGTTTGA-3;

PD-1-RV, 5-GGGCAGCTGTATGATCTGGAA-3;

LAG-3-FW, 5-CTACAACTCACCGCGTCATTT-3;

LAG-3-RW; 5-GCTCCAGACCCAGAACCTT-3;

CXCR3-FW, 5-GCCTTCCTGCTGGCTTGTAT-3;

CXCR3-RW; 5-TAGCTGCAGTACACGCAGAG-3;

TIGIT-FW, 5-GGCATGTCGCTTCAGTCTTC-3;

TIGIT-RW; 5-CTCCCCTTGTAAATCCCACC-3;

GAPDH-FW, 5-CAACAGCAACTCCCACTCTTC-3;

GAPDH-RW, 5-GGTCCAGGGTT TCTTACTCCTT-3

### Subcutaneous tumour establishment

B16 tumour cells were taken from the log phase of in vitro growth, washed and injected into C57BL/6 J mice (2 × 10^5^ cells). Tumours were clearly visible after 1 week and grew progressively in an encapsulated fashion. Induced tumours were measured on a daily basis using a vernier calliper.

### Intracranial tumour establishment

B16 melanoma cells (1 × 10^5^ taken from the log phase of in vitro growth) were injected intracranially into 8–10-week-old C57BL/6 J mice. Live imaging was performed at the time points indicated. Mice were injected intraperitoneally with luciferin (2 ug per mouse), anaesthetized with isoflurane and scanned with an IVIS Lumina (Caliper Life Sciences). For quantitative comparisons, we used Living Image software (Caliper Life Sciences) to obtain the maximum radiance (photons per s per cm^2^ per steradian, i.e. photons s^−1^ cm^−2^ sr^−1^) over each region of interest, relative to a negative control region.

### Isolation of tumour infiltrating lymphocytes (TILs)

Tumours were harvested 24 h after the last treatment was given. Tissue was disrupted using a blade and then incubated in HBSS solution containing 200 units/ml of collagenase at 37 °C for 2 h. Tissue was then passed through a strainer and cells collected and layered onto Ficoll before centrifugation. Tumour infiltrating cells were then collected from the lymphocyte layer**.**

### Immunohistochemistry

IHC was performed on formalin-fixed paraffin-embedded tissue sections, following dewaxing, rehydration and endogenous peroxidase blocking by a 3% solution of H2O2 (Sigma) for 20 min. Antigen retrieval was performed using citrate or Tris–EDTA buffers as per antibody datasheets. Nonspecific antibody binding was blocked by incubation with either horse or goat serum (Vector). Primary antibodies were diluted 1:500 (PD-1) or 1:200 (CD8) in Antibody dilution buffer (Life Technologies #003218) and were applied for 1 h at room temp. Following two washes in TBS, ready-to-use secondary antibodies were applied (ImmPRESSTM HRP reagent kits, Vector). Sections were washed twice in TBS, and ImmPACTTM DAB (Vector) was used to detect immunolabelling. Sections were counterstained in hematoxylin and, following dehydration and clearing in xylene, were mounted in distyrene/plasticizer/xylene. Sections were scanned using Aperio, and ImageScope software (Leica Biosystems) was used to determine percentage positive cells.

### Statistical analysis

The mean and SE of each treatment group were calculated for all experiments. The number of samples is indicated in the figure legends. Unpaired Student’s *t* tests or ANOVA tests were performed using the InStat 3.0 software (GraphPad). In certain instances, statistics were done using 2-way ANOVA, or by nonparametric Mann Whitney at each time point. *P < 0.05, **P < 0.01, ***P < 0.001. Log rank tests for Kaplan–Meier survival data were performed to determine significant differences.

## Results

### Case study—elraglusib first-in-human phase 1/2 study

**Fig. 1:**
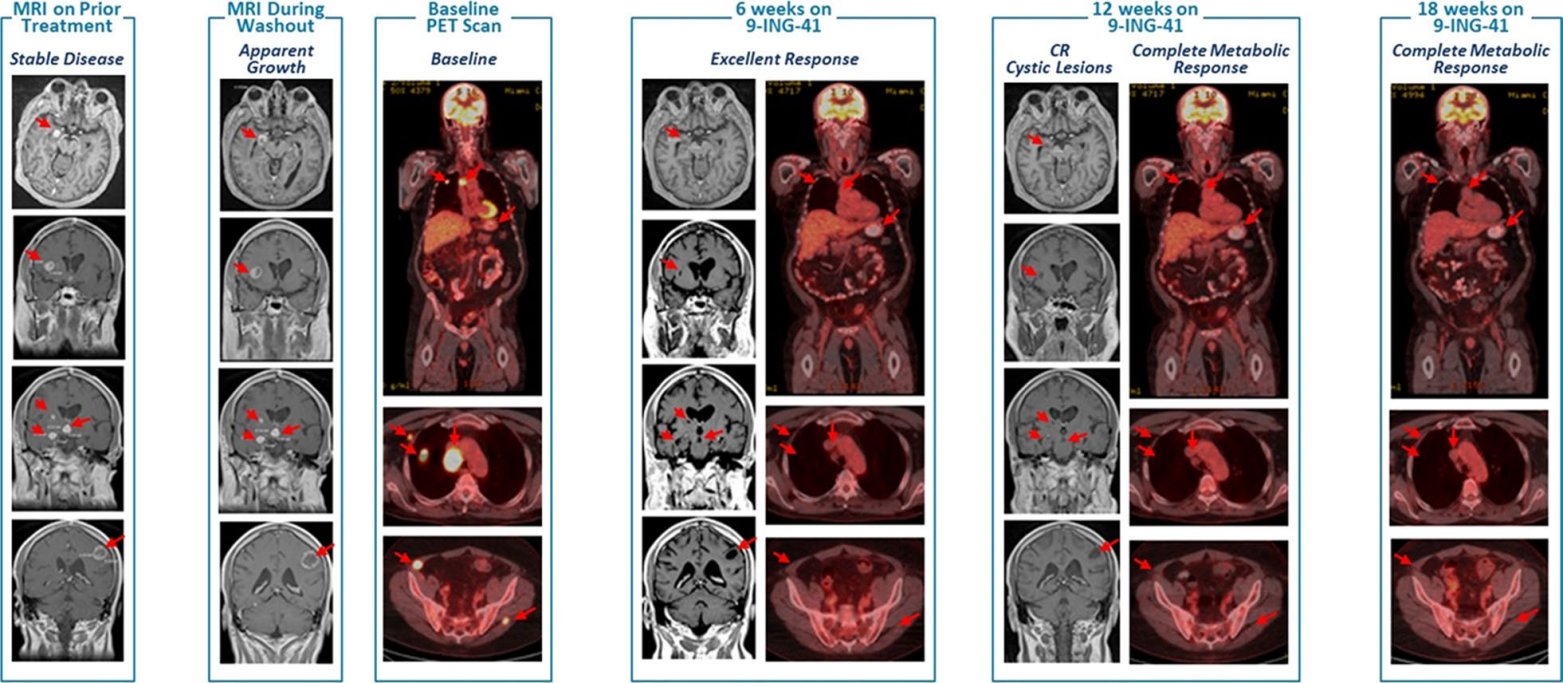
Clinical response to elraglusib monotherapy in a patient with refractory melanoma. 54-year-old male with BRAFV600E refractory melanoma and multiple sites of disease, including multiple brain lesions and progressing on anti PD-1 plus BRAF/MEK inhibitor combination. After 4-week washout brain lesions progression evident on MRI. The patient was treated with single-agent elraglusib at 5 mg/kg and within 6 weeks most sites of disease had resolved by PET, as well as significant CNS tumour shrinkage. Post-12 weeks of therapy, there was complete resolution of lesions by PET and only cystic lesions noted on brain MRI. The patient continues in CR for over 36 months

A 55-year-old white male patient, with no relevant family or personal epidemiologic or clinical history, presented with widely metastatic melanoma with involved organs including the lungs, bones, muscles, stomach, lymph nodes, pancreas and adrenal glands, as well as over 25 distinct brain lesions, was enrolled on the elraglusib 1801 first-in-human phase 1/2 study (EudraCT #:2018-003739-32; NCT #: 03678883). The patient’s melanoma was BRAF V600E mutated, and he had received multiple prior treatments including PD-1 inhibitors, CTLA-4 inhibitor, as well as BRAF/MEK inhibitor combination therapy. The most recent treatment regime prior to study entry was a combination of a PD-1 inhibitor with both BRAF and MEK inhibitors, and the patient had progressive diseases on this regimen. The patient was without treatment, “washout-period”, prior to elraglusib study entry during which time there was particularly rapid progression of brain lesions (Fig. 1). Treatment began with single-agent elraglusib at 5 mg/kg IV given twice a week on days 1 and 4. After 6 weeks, MRI showed > 30% decrease in size in all brain lesions, and PET scan demonstrated an excellent response with near complete resolution of all tumours and only residual focus of uptake in the stomach, with no new lesions. After completing 12 weeks of elraglusib treatment, MRI showed 8 cystic brain lesions with no change in size, no new lesions, and was considered a complete response by Response Assessment in Neuro-Oncology (RANO) criteria [31]. PET scan showed no areas of residual uptake, as well as resolution of uptake in the stomach, and was consistent with a complete metabolic response to therapy. The patient has never experienced any clinically significant elraglusib-attributable adverse events. The patient remains on elraglusib single-agent therapy and is now over 42 months in continuous complete response.

### Evaluation of elraglusib for pre-clinical studies in mice

**Fig. 2:**
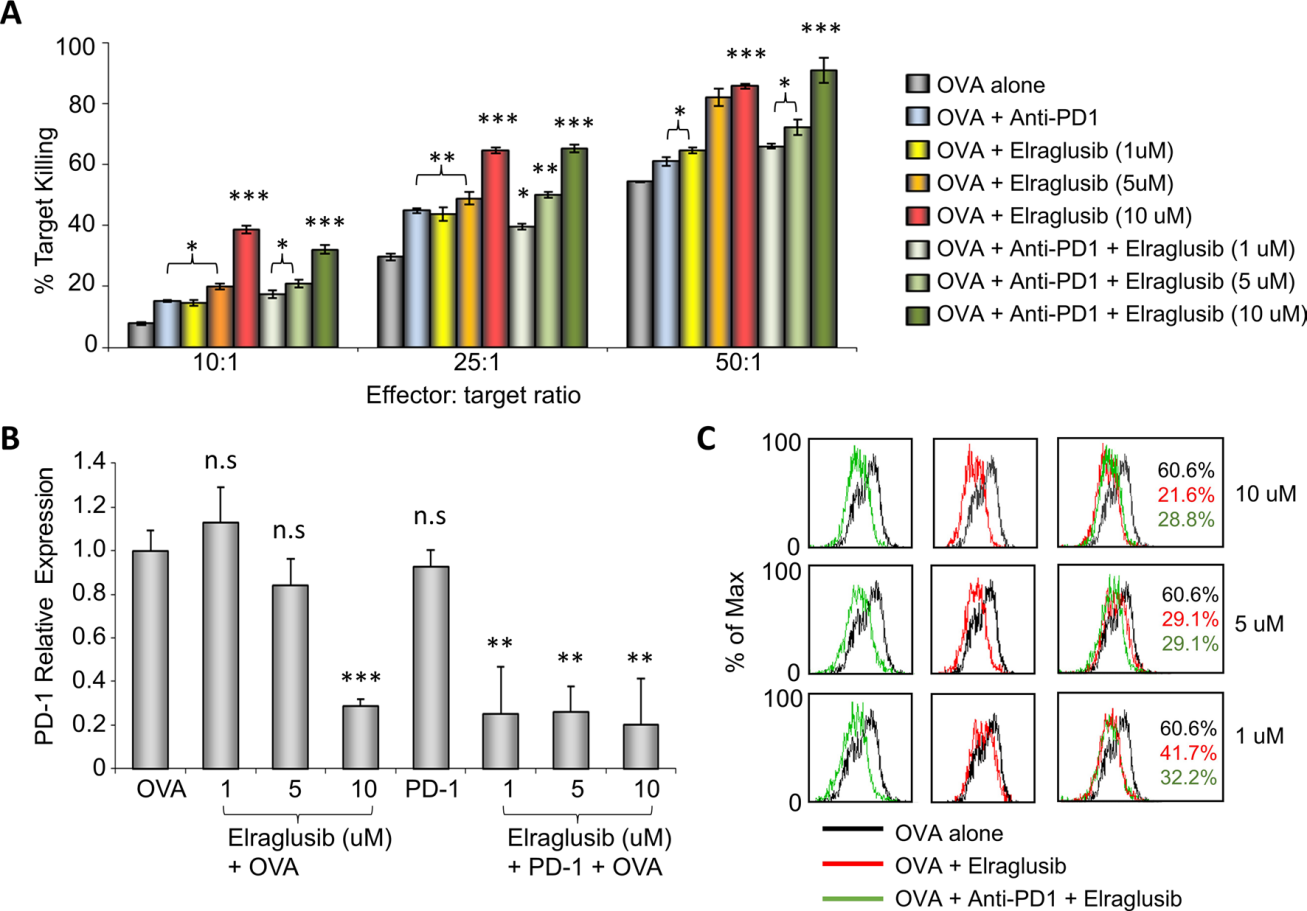
Elraglusib enhances antigen-specific CD8 + T cell cytolytic responses in vitro. **A** Cytolytic assay in response to OVA peptide in the presence of elraglusib (at the indicated concentrations) and/or anti-PD-1 mAb. **B** Quantitative real-time PCR of PD-1 transcription of CTLs treated with doses of elraglusib as indicated with/without anti-PD-1 mAb. **C** Flow cytometric profiles. Black line, non-treated CTLS; red line, with elraglusib; and green line, with elraglusib and anti-PD-1 mAb. Doses of elraglusib shown on the right. % of CTLs expressing PD-1 shown in black for non-treated cells; red for treatment with elraglusib; green for treatment with elraglusib and anti-PD-1. Statistical analysis based on triplicate values in individual experiments showing means and SDs (unpaired *t* test). *p < 0.05; **p < 0.01; ***p < 0.001; ns, no significant difference relative to controls (OVA alone). Data are represented as mean ± standard error of mean (SEM)

Previous work has shown that inactivation of GSK-3 through small-molecule inhibitors can enhance antigen-specific CD8 + CTL responses against tumour targets in vitro [9]. Here, we evaluate the use of small-molecule drug, elraglusib, to enhance these CTL responses. Initially, we examined responses of CTLs generated from OT-I transgenic mice that carry an MHC class I-restricted T cell receptor (TCR) specific for the SIINFEKL peptide of OVAlbumin (OVA_257-264_) as presented by H-2 kb. OT-I T cells were activated by OVA_257-264_ alone or in the presence of elraglusib and/or anti-PD-1 mAb for 5 days, following which the CTLs were subjected to a 4 h incubation with EL4 lymphoma target cells before lactate dehydrogenase (LDH) release was measured as an indication of cytolytic killing. EL4 cells were pulsed with OVA peptide prior to the incubation to ensure a specific response, and wells containing non-pulsed EL4 cells or effector CTLs alone were used as a control for spontaneous (background) cell death. Three different doses of elraglusib were compared in combination with or without anti-PD-1 mAb treatment. LDH release showed a significant increase in target killing in the presence of elraglusib with the concentration for optimal target death being determined as 10uM (Fig. 2A). At an effector to target ratio of 10:1, elraglusib increased the percentage of target killing from 10 to 39%, at 25:1, killing increased from 32 to 64% and at 50:1 from 59 to 86%. CTLs treated with anti-PD-1 mAb showed an increase in CTL activity compared to non-treated CTLs but had no further effect on CTLs treated with elraglusib at any of the doses used.

As predicted from previous work using GSK-3 SMIs, the upregulation of PD-1 expression due to OVA activation was dramatically reduced in the presence of elraglusib as demonstrated through PCR analysis (Fig. 2B) and flow cytometry (Fig. 2C). Although the combination of anti-PD-1 with elraglusib did not show any significant increase in cytolytic killing in vitro compared to the use of elraglusib alone, the combination did reduce PD-1 transcription at suboptimal levels (1 and 5uM) of elraglusib as seen by PCR and flow cytometry.

### Elraglusib suppresses subcutaneous B16 melanoma growth as a single agent and enhances anti-PD-1 therapy when given sequentially

**Fig. 3:**
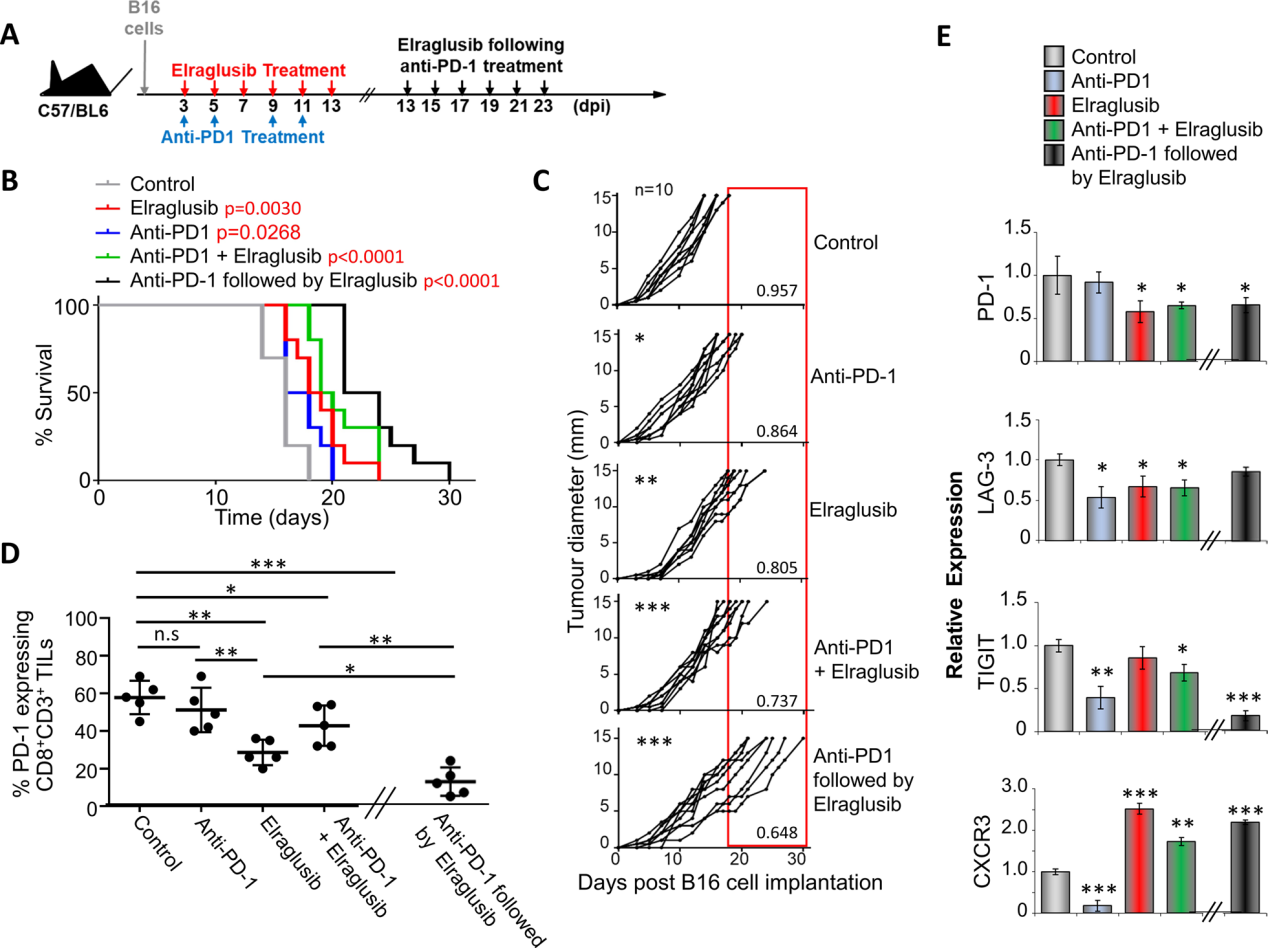
Sequential treatment of anti-PD-1 therapy followed by elraglusib significantly reduces growth of subcutaneously injected B16 melanoma. **A** Schematic representation of treatment regime. **B** Survival curves of mice with and without treatment as indicated (*n* = 10; total number of mice: 50). *P* values shown in Additional file 1: Table 1. **C** Tumour growth curves (*n* = 10 mice per condition). There was a significant difference between all groups when compared to the control. This difference was also observed when comparing the monotherapies with the sequential treatment (shown in red) but not when compared to the combined treatment. The numbers in the bottom corner indicate the average tumour growth rate represented as mm/day. **D** % of CD8 + CD3 + tumour infiltrating cells expressing PD-1 as determined by flow cytometry (*n* = 5). **E** Transcription of PD-1, LAG-3, TIGIT and CXCR3 in splenic CD8 + T cells from mice treated as shown. (B–C) Represent two pooled experiments (D-E) Data shown from one individual experiment, representative of two independent experiments. Samples taken at day 14 (after treatment with elraglusib, anti-PD-1 mAb or combined treatment) or day 24 (sequential treatment) groups are compared using unpaired *t* test. *p < 0.05, **p < 0.01, ***p < 0.001. Data are represented as mean ± SEM

As elraglusib clearly enhanced cytolytic killing by CD8^+^ CTLS in vitro, we next assessed whether elraglusib could regulate tumour growth in an in vivo setting. To do this, B16 tumour cells were injected subcutaneously into C57/BL6J mice, followed by intra-peritoneal injections of either anti-PD-1, elraglusib or a combination of the two as outlined (Fig. 3A). To mimic the findings found previously in the clinical case (Fig. 1), one group of mice received anti-PD-1 treatment and, following a 48 h break, went on to receive elraglusib treatment on days 13, 15, 17, 19, 21 and 23. Due to the aggressive nature of B16 melanoma tumour growth, it was not possible to lengthen this “break” time between treatments. Tumour growth was monitored over time, and survival was based on tumour burden, with mice killed upon tumours reaching a maximum diameter of 15 mm. Kaplan–Meier survival plots demonstrated increased survival in mice treated with elraglusib alone (50%) which was higher than that with anti-PD-1 treatment alone (30%) when compared to 0% of mice non-treated mice after 18 days post-tumour cell injection. Moreover, this survival was increased to 80% when treated with a combination of both elraglusib and anti-PD-1 Ab, and 100% when mice received sequential treatments (Fig. 3B). This increased survival was due to a decreased rate in tumour growth as can be seen from the daily tumour measurements (Fig. 3C). Tumour growth rate was measured as mm/day and demonstrated all treatments to have a significant reduction in rate. Combined treatment demonstrated a significant difference when compared to anti-PD-1 treatment alone (*p* = 0.0032) but not elraglusib treatment alone; however, sequential therapy showed a significant difference compared to elraglusib treatment alone (*p* = 0.001), PD-1 treatment alone (*p* < 0.0001) and combined treatment (*p* = 0.0248). Flow cytometry demonstrated this suppressed tumour rate to be associated with a decrease in PD-1 expression when comparing tumour infiltrating lymphocytes (TILs) from the different treatment groups. Furthermore, this reduced PD-1 expression was primarily associated with elraglusib treatment and was further reduced due to combination or sequential treatments (Fig. 3D). Tumours and spleens were harvested 24 h after the last treatment was given (day 14 for mice receiving elraglusib alone and or in combination with anti-PD-1, the samples for the sequential treatment group were taken on day 24; although there may be differences due to development of the tumour itself, it was decided to compare samples after completing treatment; to take samples from the sequential group at day 14 would not take into account the second part of treatment and would resemble those receiving anti-PD-1 alone and to take all samples at day 24 was not viable due to low survival and any effects due to the treatment may no longer be apparent).

This effect on PD-1 expression was shown further through qPCR of isolated spleen T cells showing a marked decrease in *pdcd1* transcription (Fig. 3E). Furthermore, this was accompanied by a decrease in other co-inhibitory checkpoint molecules LAG-3 and TIGIT. C-X-C Motif Chemokine Receptor 3 (CXCR3) on the other hand showed an increase in transcription due to the presence of elraglusib treatment.

### Elraglusib as a single agent or in combination with anti-PD-1 therapy significantly supresses the growth of intracranial B16 melanoma

**Fig. 4:**
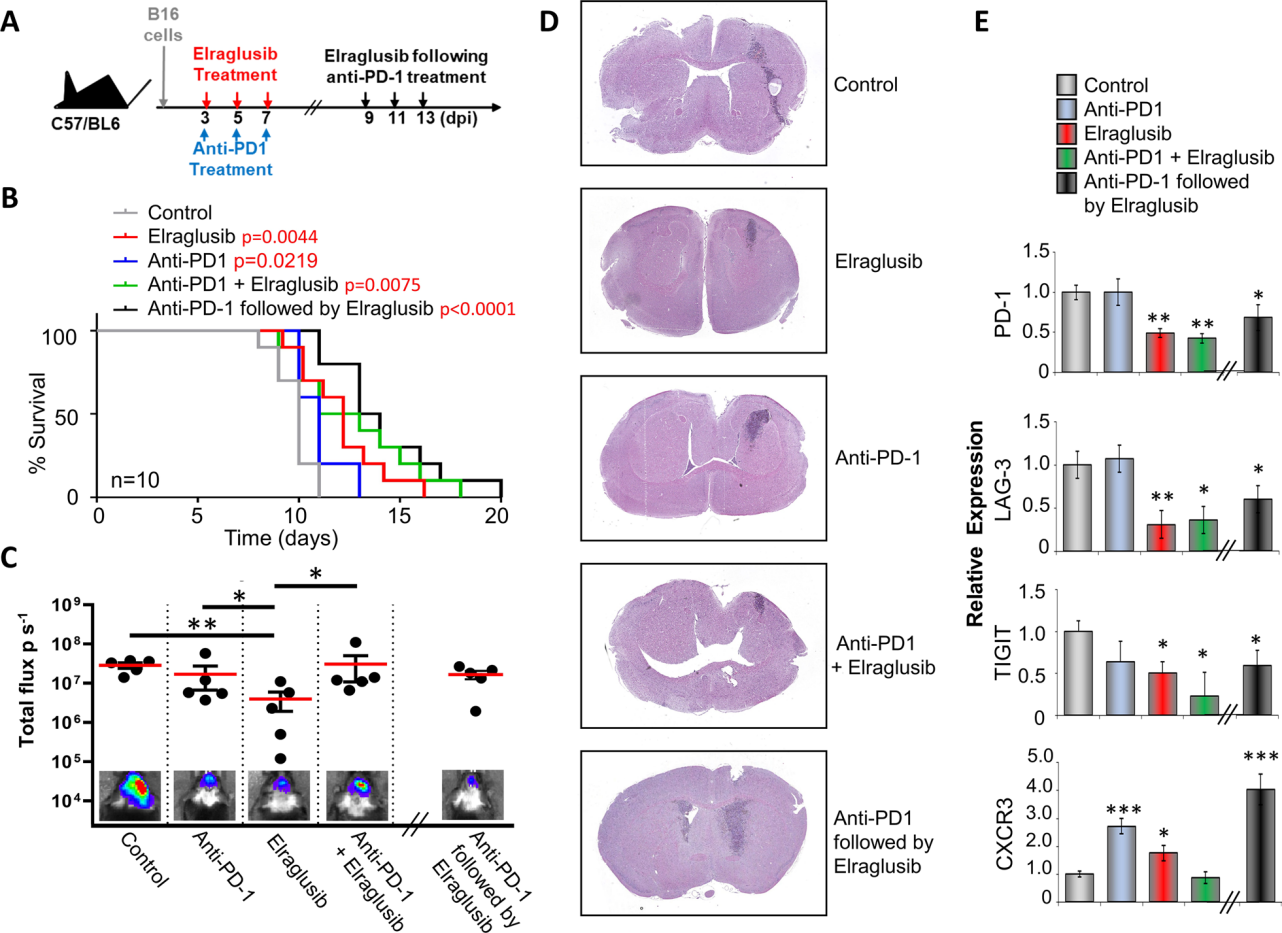
Sequential treatment of anti-PD-1 therapy followed by elraglusib significantly reduces growth of intracranial B16 melanoma. **A** Schematic representation of treatment regime. **B** Survival curves of mice with and without treatment as indicated (*n* = 10; total number of mice: 50). *P* values shown in Additional file 2: Table 2. **C** B16 metastasis at day 8 (treatments on the left) or day 14 (sequential treatment on the right) with total flux (photons/second) values depicted in the histogram. Luminescent image (bottom) shows 1 example of each group (*n* = 5 mice per condition). **D** H&E staining of brains taken at day 8 (after treatment with DMSO, elraglusib, anti-PD-1 mAb or combined treatment) or day 14 (sequential treatment) **E** Transcription of PD-1, LAG-3, TIGIT and CXCR3 in splenic CD8 + T cells from mice treated as shown. Spleens were taken at day 8 (after treatment with DMSO, elraglusib, anti-PD-1 mAb or combined treatment) or day 14 (sequential treatment). (**B**–**C**) Represent two pooled experiments (**E**) Data shown from one individual experiment, representative of two independent experiments. Groups are compared using unpaired *t* test. *p < 0.05, **p < 0.01, ***p < 0.001. Data are represented as mean ± SEM

**Fig. 5:**
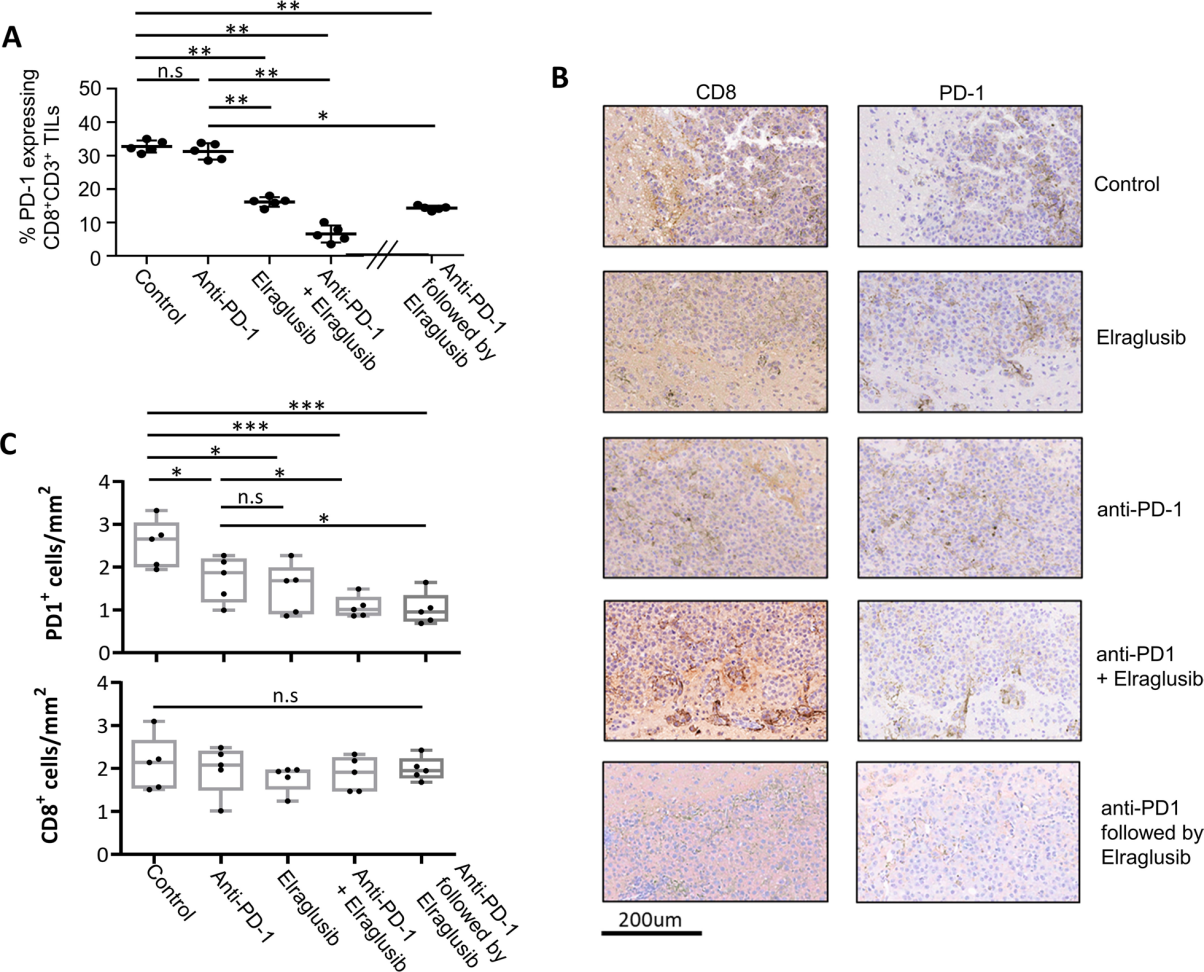
Tumour suppression by Elraglusib is associated with low numbers of PD-1 expressing tumour infiltrating T cells. **A** % of CD3 + CD8 + tumour infiltrating cells expressing PD-1 as determined by flow cytometry (*n* = 5). **B** Immunohistochemistry of brain tissue sections from B16 tumour bearing mice. Same samples as in Fig. 4D, taken at day 8 (after treatment with elraglusib, anti-PD-1 mAb or combined treatment) or day 14 (sequential treatment) as indicated to the right intra-tumoural areas are shown following staining for (left) CD8 + and (right) PD-1 + expression. **C** depicts number of positive cells quantified from 5 different areas using ImageScope software. Data representative of two independent experiments. Groups compared using unpaired *t* test. ***P < 0.0001; **P < 0.001; *P < 0.01; ns, no significant difference relative to controls

Melanoma is well known to spread to various locations in the body, particularly to the brain, and the B16 melanoma mouse model has been previously utilized to demonstrate this [32]. Here, we injected 2 × 10^5^ fLuc tagged B16 cells intracranially into C57/BL6 mice, and following the treatment regime shown (Fig. 4A), we tracked tumour growth over time using IVIS live imaging. Survival was based on when mice started to show initial symptoms of a brain tumour, i.e. severe lethargy, weight loss, disorientation. Non-treated mice showed a maximum of 11 days survival following tumour injection which was increased to 60% of mice treated with elraglusib alone, 20% with PD-1 Ab alone and 50% when treated with a combination of both elraglusib and anti-PD-1 Ab. However, this survival was increased further (80%) when mice were sequentially treated with PD-1 Ab followed by elraglusib (Fig. 4B). At day 8 (or day 14 for the sequential treatment group) 24 h after the last treatment was given, mice were injected intraperitoneally with luciferin and scanned by IVIS Lumina imaging (Fig. 4C). Total flux (photons per second) was measured showing a clear reduction in luciferase signal in response to treatment with elraglusib. This is representative of the haematoxylin and eosin (H&E) performed at these time points (Fig. 4D). The control (DMSO vehicle-treated group) shows the intracranial tumour to have a rapid growth rate with an invasive growth pattern as seen by the heavy staining. Both monotherapies show a more compact tumour area with the elraglusib showing a clear decrease in tumour size. Although the combination of elraglusib with anti-PD-1 did not appear to significantly reduce the signal as seen by IVIS, the hematoxylin and eosin (H&E) staining does show a clear decrease in tumour size which aligns with the increase in survival as shown in Fig. 4B. This is similar for the sequential treatment; in this case the tumour is more spread than in the combined treatment; however, the intensity of the staining is lower. It should also be noted that this sample was taken at day 14 which is 6 days later than the other samples, which clearly supports the increased survival shown in Fig. 4A. Quantitative PCR (Fig. 4E) on these samples revealed decreased transcription of checkpoint molecules, PD-1, LAG-3 and TIGIT as seen previously in the subcutaneous model (Fig. 3E), which was accompanied by an increase in CXCR3 transcription. This reduced expression of PD-1 was also seen in CD8 + TILs isolated from brain tumours in response to all 3 treatments incorporating elraglusib as demonstrated by flow cytometry (Fig. 5A). This was further confirmed through immunohistochemistry of sections taken from the samples in Fig. 4D. Staining showed CD8 + and PD-1 + cells to be scattered throughout the tumour region (Fig. 5B); however, significantly decreased numbers of PD-1 + cells were seen in all treatments (Fig. 5C), although there was no significant change in the number of CD8 + cells.

### Elraglusib significantly improves survival when tumour burden is spread over two locations

**Fig. 6:**
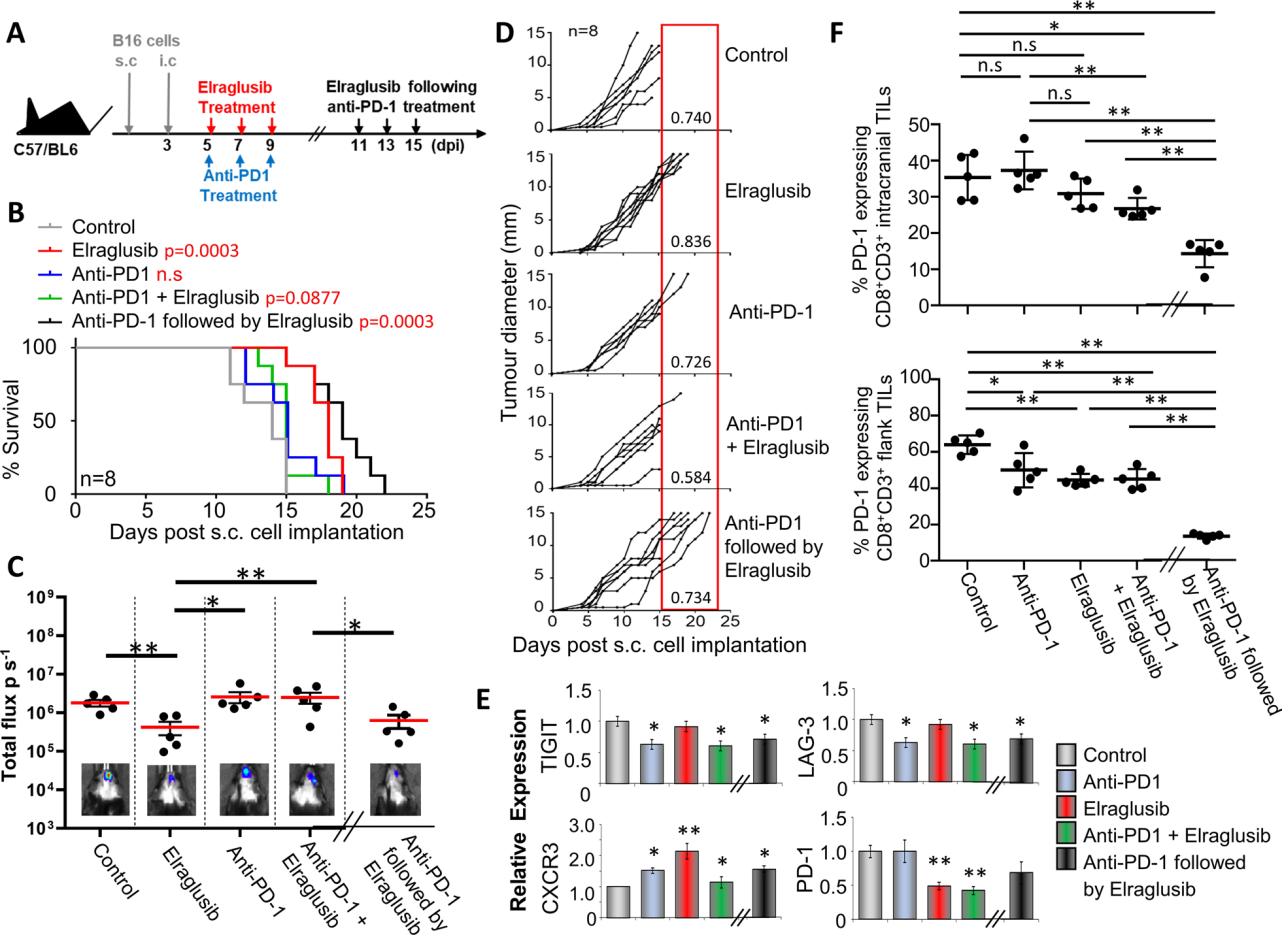
Sequential treatment of anti-PD-1 therapy followed by Elraglusib significantly reduces growth of B16 melanoma. **A** Schematic representation of treatment regime. **B** Survival curves of mice with and without treatment as indicated (*n* = 8; total number of mice: 40). *P* values shown in Additional file 3: Table 3. **C** B16 metastasis at day 10 (treatments on the left) or Day 16 (sequential treatment on the right) with total flux (photons/second) values depicted in the histogram. Luminescent image (bottom) shows 1 example of each group (*n* = 5 mice per condition). **D** Tumour growth curves (*n* = 8 mice per condition). **E** Transcription of PD-1, LAG-3, TIGIT and CXCR3 in splenic CD8 + T cells from mice treated as shown. **F** % of CD3 + CD8 + tumour infiltrating cells expressing PD-1 as determined by flow cytometry (*n* = 5). (B-D) Represent two pooled experiments (E–F) Data shown from one individual experiment, representative of two independent experiments. Samples taken at day 10 (treatments on the left) or day 16 (sequential treatment on the right). Groups are compared using unpaired *t* test. *p < 0.05, **p < 0.01, ***p < 0.001. Data are represented as mean ± SEM

In human melanoma, the majority of brain metastases coincide with extracranial metastases, usually present in the skin; to mimic this we utilized a 2-stage model as demonstrated by Taggart et al. [39]. In this model, mice were initially given a subcutaneous tumour cell injection, followed by an intracranial tumour cell injection 3 days later. The kinetics of this and the therapy given were confirmed by the previous intracranial and subcutaneous studies and are summarized in the schedule shown (Fig. 6A).

Survival was based on when mice started to show initial symptoms of a brain tumour, i.e. severe lethargy, weight loss, disorientation, or when the subcutaneous tumour reached 15 mm in diameter. The previous experiments in Figs. 3 and 4 determined a mean survival time of 16 days and 10 days for animals receiving subcutaneous or intracranial tumours alone, respectively. Therefore, staggering the implantations allowed for quantification of intracranial tumour-dependent survival. Survival (Fig. 6B) is depicted as days post the initial subcutaneous tumour injection and therefore when comparing to the intracranial injections alone a difference of − 3 days should be taken into consideration. Non-treated mice showed a maximum of 15 days survival following subcutaneous tumour injection which was increased to 25% with PD-1 Ab alone and 87.5% of mice treated with either elraglusib alone or as a sequential treatment. However, survival was only slightly increased to 12.5% when treated with a combination of both elraglusib and anti-PD-1 Ab. This demonstrated the single agents in this case to be most effective, particularly elraglusib, and that giving simultaneous although slightly better than no treatment was detrimental compared to giving single or sequential treatment. Further to this, the two different locations were assessed for the impact of the treatment on tumour growth. Live imaging (Fig. 6C) at day 10 (day 16 for those receiving sequential treatment) post-subcutaneous tumour injection showed the intracranial signal to be significantly lower in both the elraglusib and the sequential treatment group supporting the increased survival of these groups.

Although all mice were subjected to two tumours, it can be seen from the growth curves (Fig. 6D) that survival of the majority of mice receiving DMSO (vehicle control) or PD-1 therapy either as a single agent or simultaneously with elraglusib treatment was determined by the intracranial tumour, as the subcutaneous tumours in these animals did not reach the maximum 15 mm in diameter. However, the majority of animals receiving elraglusib treatment alone (5 out of 8 mice) or as a sequential treatment (7 out of 8 mice) survived the intracranial tumour and succumbed to the subcutaneous tumour growth. 62.5% of animals (5 out of 8) receiving sequential treatment were surviving at day 19, which was 16 days following intracranial injection. This survival was markedly increased compared to mice receiving only the intracranial injection (Fig. 4B), which demonstrated 30% survival at day 16. A similar, albeit lower, trend was seen in those receiving elraglusib treatment alone (25% versus 10%).

Quantitative PCR (Fig. 6E) on these samples revealed decreased transcription of checkpoint molecules, PD-1, LAG-3 and TIGIT, accompanied by an increase in CXCR3 transcription. This reduced expression of PD-1 was also seen in both CD8 + TILs isolated from intracranial and subcutaneous tumours in response to all 4 treatments as demonstrated by flow cytometry (Fig. 6F).

## Discussion

Our previous studies have demonstrated that targeting GSK-3 through siRNA knockdown, small-molecule inhibitors and genetic knockdown mouse models increases survival in tumour models including EL4 lymphoma and B16 melanoma [10, 18]. Elraglusib is the first GSK-3β inhibitor to demonstrate single-agent clinical activity in patients with refractory tumours. The case study reported here is from a patient in an ongoing clinical trial and provided rationale for this work, which indicates elraglusib’s potential utility in combination strategies with checkpoint blockade immunotherapy. In patients on this ongoing study, durable clinical responses to single-agent elraglusib have been observed, including in patients with refractory acute T cell leukaemia/lymphoma and malignant gliomas, with a favourable adverse event profile [28, 29].

These clinical data validate the pre-clinical observations that elraglusib, on evaluation in a plethora of models consistently, showed a tumour-suppressive effect as both single agent and in combination with other cytotoxic chemotherapy agents. Here, we demonstrate that elraglusib monotherapy suppresses melanoma growth, and we sought to determine if this was secondary to effects on immune checkpoint molecules, particularly PD-1 expression. Moreover, we evaluated if a combined approach with PD-1 therapy could enhance this suppressive activity. Our previous data have shown GSK-3β inhibition to be as effective as PD-1 therapy, but did not demonstrate any beneficial effect from combining the two treatments [18]. Here, we show that elraglusib treatment resulted in decreased expression of PD-1, alongside two other checkpoint molecules: TIGIT and LAG-3. Further to this, there was a clear upregulation of the chemokine receptor, CXCR3. Expression of CXCR3 has been proposed as a predictor of clinical outcomes with atezolizumab (anti-PDL-1 mAb therapy) [33] and is highly expressed on primed effector T cells, playing an important role in T cell recruitment and function [34, 35]. CXCR3 is involved in T cell recruitment, and this along with other studies suggests that GSK-3 may be involved in the migration and/or motility of T cells. This was seen previously when looking at the inhibitor SB415286 [36] and also when examining GSK-3 deficient T cells in response to tumours [37]. Here, in the 2-stage model the mice treated with elraglusib alone or sequentially show lower incidence of brain tumours compared to the other conditions. This is suggestive of an elraglusib effect on CTL trafficking from the extracranial milieu to the intracranial tumour site, eventually leading to tumour death. Furthermore, an increase in survival after sequential treatment was demonstrated throughout the studies shown here, suggesting that future approaches should focus on sequential strategy rather than concurrent treatment. This led us to test different therapy sequencing, and we demonstrated that survival is not increased when elraglusib treatment is followed by anti-PD-1 mAb treatment (data not shown). This would indicate that cells may be optimally “primed” by an anti-PD-1 treatment initially, and the subsequent exposure to elraglusib enhances the cytolytic potential of CD8 + T cells and/or leads to changes in migratory/motility machinery.

In addition to T cells, other immune cells with non-MHC I-dependent mechanisms such as NK cells may impact the immune microenvironment in cancer. GSK-3β inhibition is a known strategy for maturation of NK cells [38], as exemplified by the tool compound CHIR99021 currently used in the manufacturing of NK cell therapies. Elraglusib has been demonstrated to boost NK mediated killing of colon cancer tumour cells [39].

Using the global gene computational network approach to infer potential enhancers of tumour response to anti-PD-1 therapies, Wu et al. have recently identified, in samples from patients with melanoma, GSK-3β inhibition as an approach likely to enhance anti-PD-1’s efficacy [40].

We have demonstrated that treatment of melanoma-bearing mice with the single-agent elraglusib suppresses tumour growth and increases survival. This survival is increased further when elraglusib is given after anti-PD-1 mAb treatment. This has several potential implications for current immunotherapy regimes, including possibly reducing the number of doses of anti-PD-1 mAb treatment needed for optimal response in patients receiving elraglusib. This may be a particularly important approach given the benign adverse event profile of elraglusib in patients with advanced cancers observed to date [28]. These data highlight the potential of elraglusib as an immune-modulatory agent and provide a rationale for clinical investigation of elraglusib combinations with other immune checkpoint inhibitory molecules, including those targeting PD-1, TIGIT and LAG-3. Based on these data, and evidence of clinical activity in patients with advanced pancreatic cancer, a clinical study of elraglusib, an anti-PD-1 mAb and chemotherapy is ongoing (NCT NCT05239182).

## Supplementary Information

**Additional file 1:** Table 1 shows the full list of P values calculated for data shown in figure 3 B.

**Additional file 2:** Table 2 shows the full list of P values calculated for data shown in figure 4 B.

**Additional file 3:** Table 3 shows the full list of P values calculated for data shown in figure 6 B.

## Data Availability

All data generated or analysed during this study are included in this published article and its supplementary information files. All source data are available on request to the corresponding author.

## Abbreviations

CXCR3: C-X-C chemokine receptor type 3
GSK-3β: Glycogen Synthase Kinase-3β
PD-1: Programmed cell death protein 1
TIGIT: T cell immunoreceptor with immunoglobulin and ITIM domains
LAG-3: Lymphocyte activation gene-3
mAb: Monoclonal antibody

## References

[1] Embi N, Rylatt DB, Cohen P (1980). Glycogen synthase kinase-3 from rabbit skeletal muscle. Separation from cyclic-AMP-dependent protein kinase and phosphorylase kinase. European J Biochem/FEBS.

[2] Eldar-Finkelman H, Martinez A (2011). GSK-3 inhibitors: preclinical and focus on CNS. Front Mol Neurosci.

[3] Frame S, Cohen P (2001). GSK3 takes centre stage more than 20 years after its discovery. Biochem J.

[4] Woodgett JR (1990). Molecular cloning and expression of glycogen synthase kinase-3/factor A. EMBO J.

[5] Doble BW, Woodgett JR (2003). GSK-3: tricks of the trade for a multi-tasking kinase. J Cell Sci.

[6] Rayasam GV, Tulasi VK, Sodhi R, Davis JA, Ray A (2009). Glycogen synthase kinase 3: more than a namesake. Br J Pharmacol.

[7] Sutherland C (2011). What Are the bona fide GSK3 Substrates?. Int J Alzheimers Dis.

[8] Ohteki T, Parsons M, Zakarian A, Jones RG, Nguyen LT, Woodgett JR (2000). Negative regulation of T cell proliferation and interleukin 2 production by the serine threonine kinase GSK-3. J Exp Med.

[9] Rudd CE, Chanthong K, Taylor A (2020). Small molecule inhibition of GSK-3 specifically inhibits the transcription of inhibitory co-receptor LAG-3 for enhanced anti-tumor immunity. Cell Rep.

[10] Taylor A, Harker JA, Chanthong K, Stevenson PG, Zuniga EI, Rudd CE (2016). Glycogen synthase kinase 3 inactivation drives T-bet-mediated downregulation of co-receptor PD-1 to enhance CD8(+) cytolytic T cell responses. Immunity.

[11] Taylor A, Rudd CE (2017). Glycogen synthase kinase 3 inactivation compensates for the lack of CD28 in the priming of CD8(+) cytotoxic T-Cells: implications for anti-PD-1 immunotherapy. Front Immunol.

[12] Appleman LJ, van Puijenbroek AA, Shu KM, Nadler LM, Boussiotis VA (2002). CD28 costimulation mediates down-regulation of p27kip1 and cell cycle progression by activation of the PI3K/PKB signaling pathway in primary human T cells. J Immunol.

[13] Wood JE, Schneider H, Rudd CE (2006). TcR and TcR-CD28 engagement of protein kinase B (PKB/AKT) and glycogen synthase kinase-3 (GSK-3) operates independently of guanine nucleotide exchange factor VAV-1. J Biol Chem.

[14] Borden BA, Baca Y, Xiu J, Tavora F, Winer I, Weinberg BA (2021). The landscape of glycogen synthase kinase-3 beta genomic alterations in cancer. Mol Cancer Ther.

[15] Sahin I, Eturi A, De Souza A, Pamarthy S, Tavora F, Giles FJ (2019). Glycogen synthase kinase-3 beta inhibitors as novel cancer treatments and modulators of antitumor immune responses. Cancer Biol Ther.

[16] Walz A, Ugolkov A, Chandra S, Kozikowski A, Carneiro BA, O'Halloran TV (2017). Molecular pathways: revisiting glycogen synthase kinase-3beta as a target for the treatment of cancer. Clin Cancer Res.

[17] Hui E, Cheung J, Zhu J, Su X, Taylor MJ, Wallweber HA (2017). T cell costimulatory receptor CD28 is a primary target for PD-1-mediated inhibition. Science.

[18] Taylor A, Rothstein D, Rudd CE (2017). Small molecule drug inhibition of PD-1 transcription is an effective alternative to antibody blockade in cancer therapy. Cancer Res.

[19] Ding L, Madamsetty VS, Kiers S, Alekhina O, Ugolkov A, Dube J (2019). Glycogen synthase kinase-3 inhibition sensitizes pancreatic cancer cells to chemotherapy by abrogating the TopBP1/ATR-mediated DNA damage response. Clin Cancer Res.

[20] Hilliard TS, Gaisina IN, Muehlbauer AG, Gaisin AM, Gallier F, Burdette JE (2011). Glycogen synthase kinase 3beta inhibitors induce apoptosis in ovarian cancer cells and inhibit in-vivo tumor growth. Anticancer Drugs.

[21] Kuroki H, Anraku T, Kazama A, Bilim V, Tasaki M, Schmitt D (2019). 9-ING-41, a small molecule inhibitor of GSK-3beta, potentiates the effects of anticancer therapeutics in bladder cancer. Sci Rep.

[22] Pal K, Cao Y, Gaisina IN, Bhattacharya S, Dutta SK, Wang E (2014). Inhibition of GSK-3 induces differentiation and impaired glucose metabolism in renal cancer. Mol Cancer Ther.

[23] Ugolkov A, Gaisina I, Zhang JS, Billadeau DD, White K, Kozikowski A (2016). GSK-3 inhibition overcomes chemoresistance in human breast cancer. Cancer Lett.

[24] Ugolkov A, Qiang W, Bondarenko G, Procissi D, Gaisina I, James CD (2017). Combination treatment with the GSK-3 inhibitor 9-ING-41 and CCNU cures orthotopic chemoresistant glioblastoma in patient-derived xenograft models. Transl Oncol.

[25] Ugolkov AV, Bondarenko GI, Dubrovskyi O, Berbegall AP, Navarro S, Noguera R (2018). 9-ING-41, a small-molecule glycogen synthase kinase-3 inhibitor, is active in neuroblastoma. Anticancer Drugs.

[26] Wu X, Stenson M, Abeykoon J, Nowakowski K, Zhang L, Lawson J (2019). Targeting glycogen synthase kinase 3 for therapeutic benefit in lymphoma. Blood.

[27] Karmali R, Chukkapalli V, Gordon LI, Borgia JA, Ugolkov A, Mazar AP (2017). GSK-3beta inhibitor, 9-ING-41, reduces cell viability and halts proliferation of B-cell lymphoma cell lines as a single agent and in combination with novel agents. Oncotarget.

[28] Odia Y, Cavalcante L, Safran H, Powell SF, Munster PN, Ma WW (2022). Malignant glioma subset from actuate 1801: Phase I/II study of 9-ING-41, GSK-3beta inhibitor, monotherapy or combined with chemotherapy for refractory malignancies. Neurooncol Adv.

[29] Hsu A, Huntington KE, De Souza A, Zhou L, Olszewski AJ, Makwana NP (2022). Clinical activity of 9-ING-41, a small molecule selective glycogen synthase kinase-3 beta (GSK-3beta) inhibitor, in refractory adult T-Cell leukemia/lymphoma. Cancer Biol Ther.

[30] Wacker MJ, Godard MP (2005). Analysis of one-step and two-step real-time RT-PCR using SuperScript III. J Biomol Tech JBT.

[31] Wen PY, Macdonald DR, Reardon DA, Cloughesy TF, Sorensen AG, Galanis E (2010). Updated response assessment criteria for high-grade gliomas: response assessment in neuro-oncology working group. J Clin Oncol.

[32] Taggart D, Andreou T, Scott KJ, Williams J, Rippaus N, Brownlie RJ (2018). Anti-PD-1/anti-CTLA-4 efficacy in melanoma brain metastases depends on extracranial disease and augmentation of CD8(+) T cell trafficking. Proc Natl Acad Sci USA.

[33] Iwai T, Sugimoto M, Patil NS, Bower D, Suzuki M, Kato C (2021). Both T cell priming in lymph node and CXCR3-dependent migration are the key events for predicting the response of atezolizumab. Sci Rep.

[34] Groom JR, Luster AD (2011). CXCR3 in T cell function. Exp Cell Res.

[35] Shimizu K, Yamasaki S, Shinga J, Sato Y, Watanabe T, Ohara O (2016). Systemic DC activation modulates the tumor microenvironment and shapes the long-lived tumor-specific memory mediated by CD8+ T Cells. Cancer Res.

[36] Taylor A, Rudd CE (2020). Glycogen synthase kinase 3 (GSK-3) controls T-cell motility and interactions with antigen presenting cells. BMC Res Notes.

[37] Steele L, Mannion AJ, Shaw G, Maclennan KA, Cook GP, Rudd CE (2021). Non-redundant activity of GSK-3alpha and GSK-3beta in T cell-mediated tumor rejection. iScience.

[38] Cichocki F, Valamehr B, Bjordahl R, Zhang B, Rezner B, Rogers P (2017). GSK3 inhibition drives maturation of NK cells and enhances their antitumor activity. Cancer Res.

[39] Huntington KE, Zhang S, Carneiro BA, El-Deiry WS (2021). Abstract 2676: GSK3β inhibition by small molecule 9-ING-41 decreases VEGF and other cytokines, and boosts NK and T cell-mediated killing of colorectal tumor cells. Cancer Res.

[40] Wu CC, Wang YA, Livingston JA, Zhang J, Futreal PA (2022). Prediction of biomarkers and therapeutic combinations for anti-PD-1 immunotherapy using the global gene network association. Nat Commun.

