# Supplementary figures and images for: Elraglusib (9-ING-41), a selective small-molecule inhibitor of glycogen synthase kinase-3 beta, reduces expression of immune checkpoint molecules PD-1, TIGIT and LAG-3 and enhances CD8+ T cell cytolytic killing of melanoma cells
Source: J Hematol Oncol. 2022 Sep 14;15:134. doi: 10.1186/s13045-022-01352-x (PMC9472445; doi:10.1186/s13045-022-01352-x)

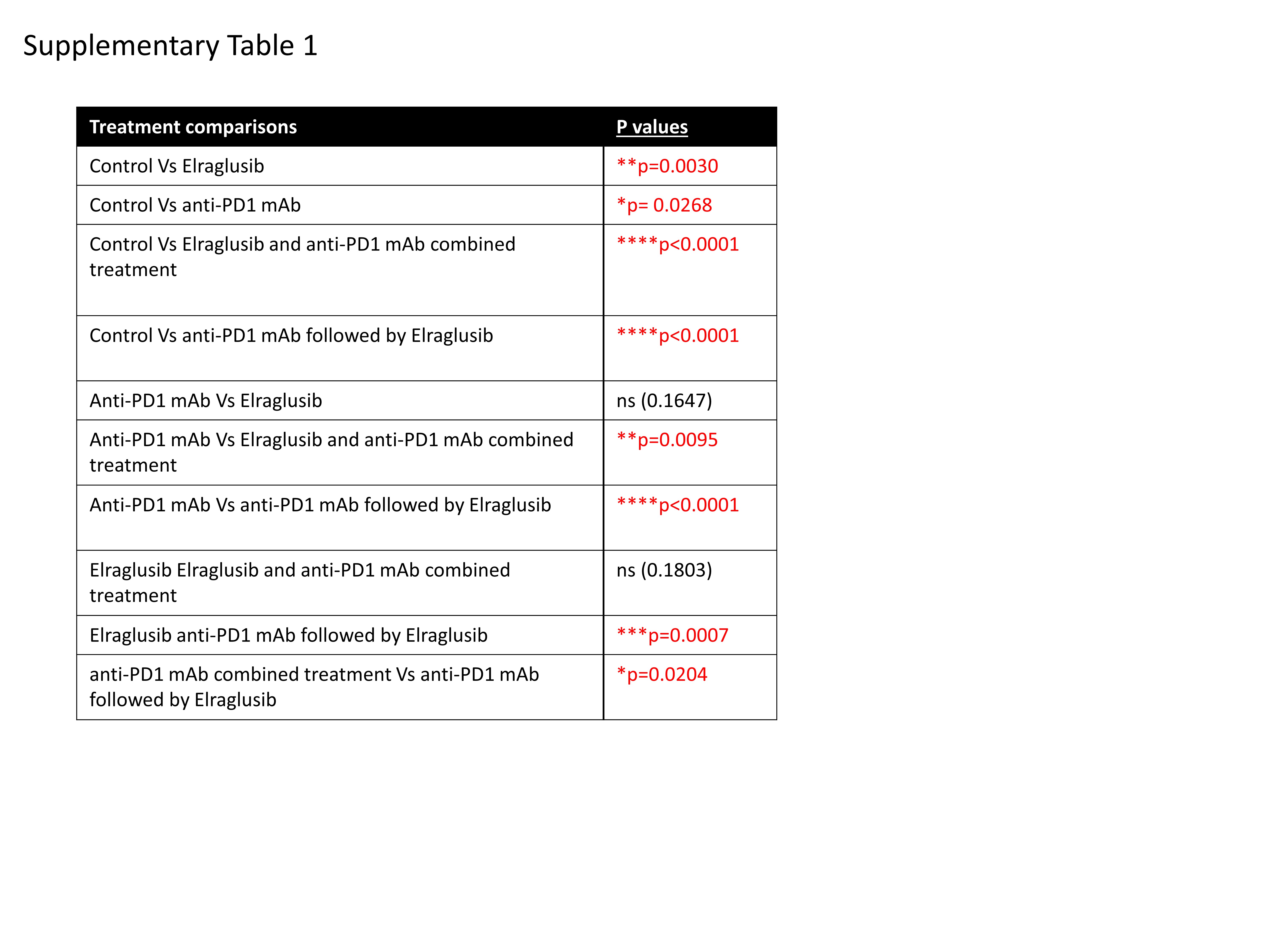

Supplement: Supplementary file 1 — Additional file 1: Table 1 shows the full list of P values calculated for data shown in figure 3 B. [file 13045_2022_1352_MOESM1_ESM.jpg]

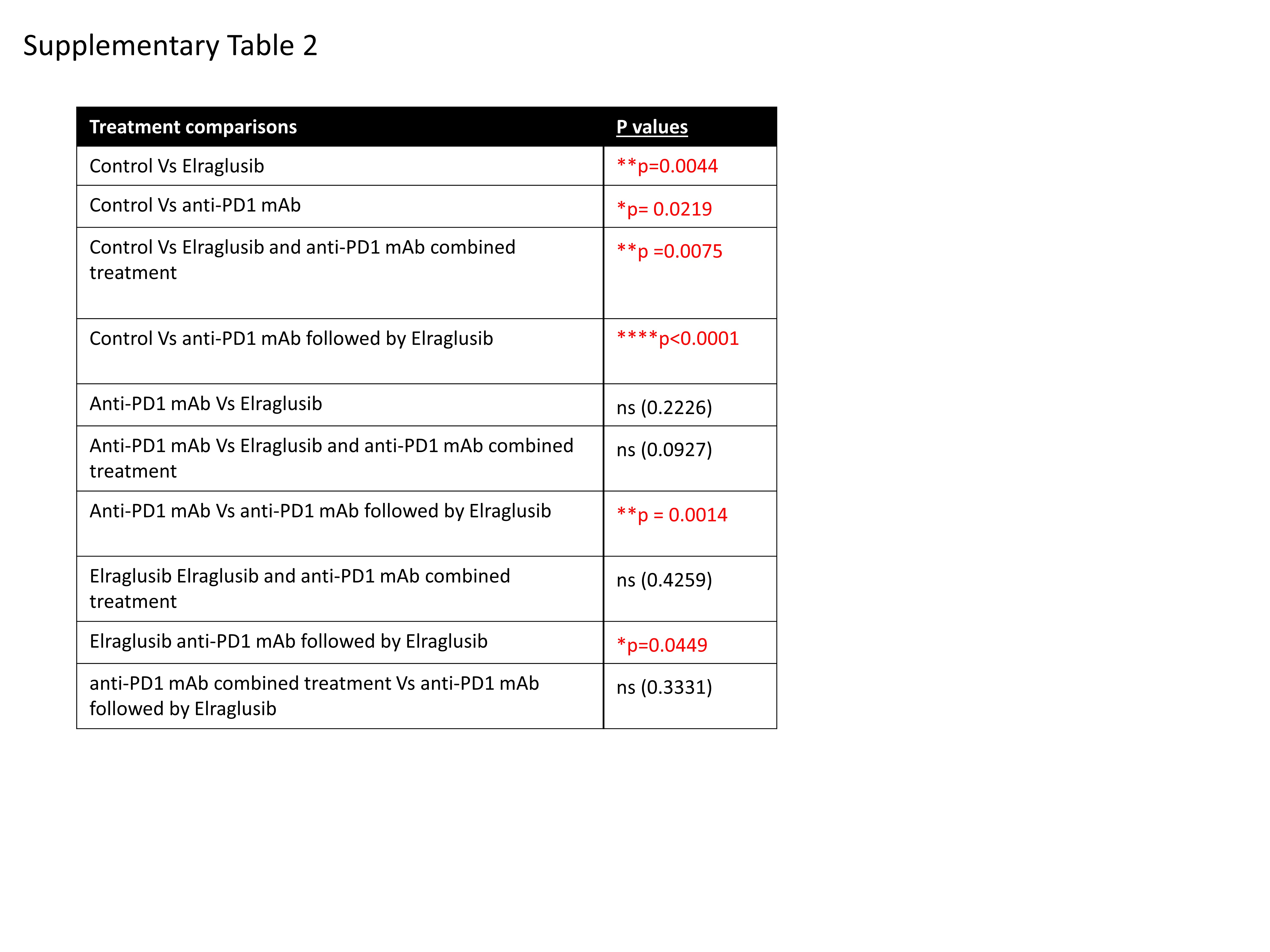

Supplement: Supplementary file 2 — Additional file 2: Table 2 shows the full list of P values calculated for data shown in figure 4 B. [file 13045_2022_1352_MOESM2_ESM.jpg]

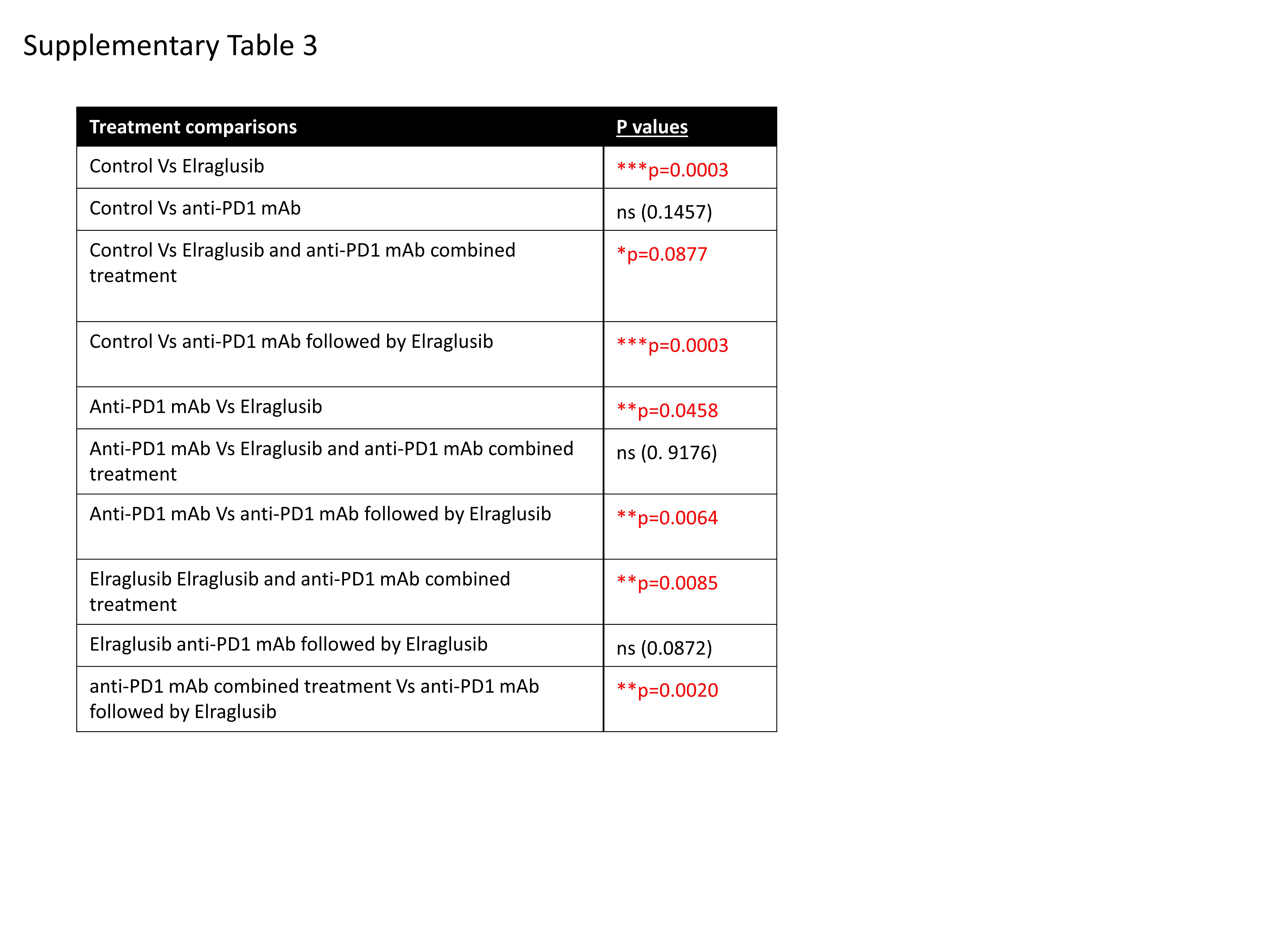

Supplement: Supplementary file 3 — Additional file 3: Table 3 shows the full list of P values calculated for data shown in figure 6 B. [file 13045_2022_1352_MOESM3_ESM.jpg]
